# Evaluation of Matrix Metalloproteinase 9 Serum Concentration as a Biomarker in Malignant Mesothelioma

**DOI:** 10.1155/2019/1242964

**Published:** 2019-05-02

**Authors:** Danijela Štrbac, Katja Goričar, Vita Dolžan, Viljem Kovač

**Affiliations:** ^1^Institute of Oncology Ljubljana, Zaloška 2, 1000 Ljubljana, Slovenia; ^2^University of Ljubljana, Faculty of Medicine, Institute of Biochemistry, Pharmacogenetics Laboratory, Vrazov trg 2, 1000 Ljubljana, Slovenia

## Abstract

**Background:**

Malignant mesothelioma (MM) is a rare, but fatal disease with few treatment options. The diagnosis and treatment response are challenging in MM. Therefore, the search for novel diagnostic and prognostic biomarkers is ongoing. The aim of our study was to investigate matrix metalloproteinase 9 (MMP9) as a potential serum biomarker of treatment response and survival in MM. We also investigated the influence of genetic polymorphisms on MMP9 serum levels.

**Methods:**

We included 110 patients with MM that have been previously genotyped for common *MMP9* polymorphisms. Serum samples were collected before treatment, at the end of chemotherapy, and at the time of progression. MMP9 serum levels were measured using enzyme-linked immunosorbent assay kits. The role of serum MMP9 and *MMP9* polymorphisms in treatment response was determined using the nonparametric tests and logistic or Cox regression.

**Results:**

Median serum MMP9 was 706.7 (499.6-1224.9) ng/ml before treatment, 440.5 (255.9-685.2) ng/ml after chemotherapy, and 502.8 (307.2-851.4) ng/ml at disease progression. After chemotherapy, 87 (79.8%) patients had lower serum MMP9, with the median change of -286.3 (-607.3 to -70.2) ng/ml (*P* < 0.001). At disease progression, 47 (65.3%) patients had lower serum MMP9 compared to pretreatment values, with the median change of -163.7 (-466.6 to 108.6) ng/ml (*P* = 0.001). Patients with higher performance status had higher serum MMP9 before treatment (*P* = 0.010). Among investigated polymorphisms, only rs17576 was associated with serum MMP9 levels before treatment (*P* = 0.041).

**Conclusion:**

Median serum MMP9 levels differed significantly before and after treatment of MM, but failed to reach significance as a standalone biomarker. The contribution of MMP9 serum levels and *MMP9* polymorphisms to a composite diagnostic and prognostic biomarker should be further tested.

## 1. Introduction

Malignant mesothelioma (MM) is a rare but fatal disease, with limited treatment options; however, new targeted therapies are emerging [1]. In the recent years, extensive efforts have been put into finding novel treatment options, as well as novel diagnostic, prognostic, and predictive biomarkers [2]. An ongoing search for the last thirty years has identified mesothelin (soluble mesothelin-related peptides, SMRP) as a promising clinically relevant biomarker for monitoring the response and disease progression in MM [3]. SMRP in pleural fluid has been suggested to distinguish pleural mesothelioma from pleural metastasis and therefore aid in the diagnosis of MM [4, 5]. Additional serum peptides, such as survivin and fibulin-3, have been investigated in an effort to find new biomarkers that would further support the diagnostic and treatment process in MM [6, 7]. Serum survivin decreased significantly with good response to treatment [6], while higher serum levels of fibulin-3 were found in patients that progressed within 18 months of treatment [7].

As none of these serum biomarkers demonstrated a high enough sensitivity as a standalone marker in mesothelioma, several attempts were made to improve the sensitivity and specificity [8–10]. For example, single nucleotide polymorphisms (SNPs) in the mesothelin gene (*MSLN*) could improve the prognostic value of mesothelin [11–13]. On the other hand, additional serum markers were studied. A study that investigated SMRP as well as matrix metalloproteinase 9 (MMP9) among several potential biomarkers concluded that the combination of radiographic findings and blood markers could be used to stratify the risk of MM in asbestos-exposed population [14]. Furthermore, MMP9 overexpression in correlation with MSLN overexpression showed increased tumor invasion and decreased survival in MM patients [15].

MMP9 was therefore investigated as a potential biomarker in MM, but its association with clinical outcome has not been elucidated in MM. There are substantial data of biological function of MMP9 in all thoracic malignancies. MMP9 is involved in turnover and remodeling of the extracellular matrix in normal respiratory epithelial growth, suggesting a potential role in carcinogenesis. Healthy lung tissue in itself has an intrinsic, basal expression of MMP9. The stromal cells overexpress MMP9 in inflammatory disease, albeit with a greater expression in tumor cells [15, 16]. However, MMP9 and MMP2 as potential biomarkers in MM did not reach diagnostic value as potential standalone biomarkers and more studies are needed to evaluate the role of soluble MMP9 in MM. We have shown that *MMP9* rs2250889 CG genotype carriers had significantly shorter overall survival (OS) and time to progression (TTP), while *MMP9* rs20544 CT allele carriers had longer OS [17, 18].

These results led us to further investigate if these polymorphisms influence MMP9 serum levels. Furthermore, we have assessed the predictive values of MMP9 serum levels in MM patients before and after treatment as well as at disease progression.

## 2. Patients and Methods

### 2.1. Patients

Patients with histologically proven pleural or peritoneal mesothelioma diagnosed and treated between 2007 and 2016 were included in this retrospective study. The patients were diagnosed at the University Clinic Golnik and University Clinic Ljubljana, Department of Thoracic Surgery. Most of the patients were treated and followed up at the Institute of Oncology, Ljubljana. Patients received chemotherapy according to standard protocols that consist of six cycles of gemcitabine/cisplatin (carboplatin) or pemetrexed/cisplatin (carboplatin) chemotherapy. Patients did not receive any maintenance chemotherapy.

Most patients included in the study were also participating in previous studies on pharmacogenomics of MPM treatment, at the Institute of Oncology, Ljubljana. Some of the patients were included in a parallel clinical trial AGILI (Trial registration ID: NCT01281800).

The study was approved by the Slovenian Ethics Committee for Research in Medicine and was carried out according to the Declaration of Helsinki.

### 2.2. Experimental Methods

Serum samples were collected before treatment, at the end of chemotherapy, and at the time of progression. We included in the study only MM patients that had available serum samples before treatment and at least one additional sample (at the end of chemotherapy or at disease progression or both). For samples at the end of chemotherapy, blood was sampled on the day of the last (6th) chemotherapy cycle, unless disease progression occurred before the last cycle. None of the patients received maintenance chemotherapy. Serum samples were prepared immediately after blood sampling, aliquoted, and stored at −20°C until MMP9 levels were measured. The remaining blood peripheral lymphocytes were used for DNA isolation, and all the patients have been genotyped for common *MMP2*, *MMP9*, and *MMP14* polymorphisms [17].

MMP9 serum levels were measured using enzyme-linked immunosorbent assay (ELISA) kits according to the manufacturer's instructions (Quantikine ELISA, R&D Systems, Minneapolis, USA). This assay enables detection of natural human and recombinant MMP9, both the 92 kDa Pro-MMP9 form and the active 82 kDa MMP9 form [19]. Minimum detectable dose of the assay is 0.156 ng/ml. The manufacturer of the assay reports the intra-assay coefficient of variation (CV) for MMP-9 as 1.9-2.9% and interassay CV as 6.9-7.9%. In our study, serum samples were diluted 100-fold. The standard curve was calculated using a four-parameter logistic curve fit.

### 2.3. Statistical Analyses

Continuous and categorical variables were described using median with interquartile range (25%-75%) and frequencies, respectively. The nonparametric Mann-Whitney or Kruskal-Wallis tests were used to compare distribution of continuous variables between different categories for independent samples, while the Wilcoxon test was used for related samples. Pairwise comparisons with post hoc Bonferroni corrections were used with the Kruskal-Wallis test. Spearman's rho was used to assess correlations between continuous variables. The association of serum MMP9 levels with response rate was determined using logistic regression, while survival analysis was performed using Cox regression to calculate hazard ratios (HRs) and their 95% confidence intervals (CIs). The Kaplan-Meier analysis was used to calculate the median survival and follow-up times. The additive and dominant genetic models were used in analyses assessing the association of SNPs with serum MMP9 concentration. Deviation from the Hardy-Weinberg equilibrium (HWE) was evaluated using the chi-square test. All statistical analyses were carried out using IBM SPSS Statistics, version 21.0 (IBM Corporation, Armonk, NY, USA). Haplotypes were reconstructed and analyzed using THESIAS software where the most frequent haplotype served as the reference. All statistical tests were two sided, and the level of significance was set to 0.05.

## 3. Results

In total, 110 MM patients were included in the study. Their characteristics are summarized in Table 1. All patients were treated with chemotherapy; 66 (60%) received gemcitabine/cisplatin (carboplatin) as first-line chemotherapy, 41 (37.3%) received pemetrexed/cisplatin (carboplatin), and 3 (2.7%) received other types of chemotherapy. 81 (73.6%) patients had epithelioid histological type, and 56 patients had Eastern Cooperative Oncology Group (ECOG) (50.9%) performance status of 1.

At the time of data evaluation, 95 (86.4%) of the patients experienced disease progression and 60 (54.5%) of the included patients were dead. Median TTP from the beginning of chemotherapy was 7.9 (5.4-14.7) months, OS was 21.8 (10.1-28.8) months, and follow-up time from the beginning of chemotherapy was 21.8 (13.8-74.9) months as well. Serum samples after chemotherapy were available for 109 out of 110 patients, while serum samples at disease progression were available for 72 out of 95 patients.

In ELISA assay, average intra-assay CV for our samples measured in duplicates on the same plate was 5.7%, while interassay CV was 10%. As intra-assay CV was below 10% and interassay CV was below 15%, the variation between measurements was sufficiently low.

Median serum MMP9 before treatment was 706.7 (499.6-1224.9) ng/ml, after chemotherapy 440.5 (255.9-685.2) ng/ml, and at disease progression 502.8 (307.2-851.4) ng/ml. After chemotherapy, 87 (79.8%) patients had lower serum MMP9 compared to pretreatment values, with the median change of -286.3 (-607.3 to -70.2) ng/ml (*P* < 0.001). At disease progression, serum MMP9 level was significantly lower compared to pretreatment values (*P* = 0.001). At progression, 47 (65.3%) patients had lower serum MMP9 compared to pretreatment values, with the median change of -163.7 (-466.6 to 108.6) ng/ml. On the other hand, serum MMP9 levels were statistically significantly higher at disease progression compared to values after chemotherapy (*P* = 0.006). At disease progression, only 17 (23.6%) patients had lower MMP9, while 36 (50.0%) patients had slightly higher serum MMP9 compared to values after chemotherapy, with the median change of 2.7 (0.0-372.7) ng/ml.

The impact of clinical characteristics on serum MMP9 levels is presented in Table 1. Patients with higher ECOG performance status had higher serum MMP9 before treatment (*P* = 0.010). The difference between patients with ECOG status 2 and patients with ECOG status 0 was statistically significant even after post hoc Bonferroni correction (*P* = 0.018). Serum MMP9 levels did not differ between different histological types (*P* = 0.830).

There was no significant difference in serum MMP9 levels before and after treatment in patient groups with different responses to treatment (Table 2).

Compared to MMP9 levels before chemotherapy, levels of MMP9 after chemotherapy decreased in 61 patients (81.3%) with stable or progressive disease, and in 26 (76.5%) patients with complete and partial response. Increased MMP9 levels were not associated with better response rate (OR = 1.34, 95% CI = 0.50-3.58, *P* = 0.559), even after adjustment for clinical parameters (OR = 1.65, 95% CI = 0.58-4.67, *P* = 0.345).

Genotype frequencies for investigated MMP9 SNPs are presented in Table 3. All were in agreement with HWE (*P* > 0.050).

Among the investigated SNPs, only rs17576 was associated with serum MMP9 levels before treatment (*P* = 0.041, Table 3). Carriers of two polymorphic G alleles had significantly higher serum MMP9 level compared to carriers of two wild-type A alleles (*P* = 0.013). The same trend was observed in the dominant model, but the difference failed to reach statistical significance (*P* = 0.053). Furthermore, in haplotype analysis, no significant association of *MMP9* haplotypes with serum MMP9 was observed (Supplementary Table 1).

In survival analysis, there were no statistically significant associations of serum MMP9 before treatment, after treatment, and at disease progression with TTP or OS, even after adjustment for clinical parameters (Table 4).

## 4. Discussion

In the present study, we evaluated MMP9 serum expression as an easily accessible serum marker in MM and investigated a possible clinical correlation to OS, TTP, and treatment response. Furthermore, we investigated a correlation between MMP9 serum levels and *MMP9* SNPs. We have observed two interesting associations: an association between MMP9 serum levels and ECOG performance status and between MMP9 expression and *MMP9* rs17576 polymorphism.

A statistically significant result of our analysis was a correlation between higher MMP9 serum levels before treatment and higher ECOG performance status. Patients that were fully active and able to carry on daily activities without restrictions (ECOG 0) had a significantly lower MMP9 serum concentration in comparison to the patients capable of self-care, but up and about 50% of the waking hours (ECOG 2). In a lung cancer study, comparing expression of MMP9 in normal vs. cancer tissue MMP9 expression was the highest in adenocarcinoma of greater tumor stage (III and IV). The expression and activity of MMP9 in serum as well as tissue samples correlated with histology and tumor stage, without a reference to performance status [20].

We observed a significant decrease of serum MMP9 after chemotherapy, while the levels rose again at disease progression. Our observations are in agreement with a study in lung cancer concluding that an increase in MMP9 levels after three cycles of chemotherapy was predictive for progression with 5% increase in the odds of progression for an increase of 10 ng of MMP9 [21]. Also, Qiao et al. showed that differences in serum MMP9 concentration in lung cancer patients before and after disease progression were statistically significant after one, two, and four cycles of chemotherapy [22]. Moreover, a meta-analysis on MMP9 serum concentration in lung cancer, including 26 studies, concluded that MMP9 overexpression correlated with advanced tumor stage and poor 5-year OS. This study noted a difference between the serum and tumor tissue overexpression of MMP9. An overexpression of MMP9 in tumor tissue, but not the serum, had the abovementioned influence on tumor stage and OS [23]. MMP9 has an important role in metastasis of lung adenocarcinoma that was investigated in a recent study. Their conclusion was that an elevated MMP7 and MMP9 play a role in the release of circulating tumor cells into the peripheral blood. The investigators suggested that the concentrations of MMP7 and MMP9 and the circulating tumor cell count could be used together as an effective, clinically predictive panel for lung adenocarcinoma metastasis and prognosis [24]. In other tumors, such as gastric cancer, MMP9 plasma levels correlated better with disease progression and lymph node invasion than the serum levels. Therefore, we believe that further studies are needed to determine the role of serum MMP9 as a diagnostic/prognostic marker [25].

We also set out to investigate a possible link between MMP9 serum concentrations, treatment response to chemotherapy, and an association with TTP and OS in MM patients. We observed some differences in MMP9 serum concentration in patients with complete or partial response and in patients with stable or progressive disease, but these concentration differences failed to reach statistical significance and were not associated with survival or treatment response.

Our study focused on MMP9 concentration in combination with *MMP9* polymorphisms as biomarkers in MM. We observed a large interindividual variability in MMP9 serum levels, both before treatment and at different treatment responses; therefore, we investigated the role of genetic variability in MMP9 genes. In our previous study, we established the influence of *MMP9* rs2250889 and rs20544 on TTP and OS [18]. In this study, statistically significant association was found between *MMP9* rs17576 A>G GG genotype and higher MMP9 serum levels before treatment in comparison to the wild-type *MMP9* rs17576 A>G AA genotype. This genotype showed a tendency of association with TTP and OS; however, it was not statistically significant.

Our present and our previous studies suggest that MMP9 may play a role in MM. Although MMP9 serum levels failed to reach significant associations with clinical endpoints, therefore we believe that further studies should be performed to elucidate the role of serum MMP9 as a diagnostic/prognostic marker. The limitations of our study was a relatively small sample size, due to the rarity of MM and the lack of comparative data on tumor tissue MMP9 expression levels. However, to the best of our knowledge, this is the first such longitudinal study in MM. Furthermore, we investigated both serum and genetic biomarkers in a well-characterized population of MM patients treated with the same protocols and followed up in the same institution.

Our data suggests that, rather than as standalone biomarkers, MMP9 serum expression as well as *MMP9* rs17576 A>G genotype may perform better in combination with other potential biomarkers. Further studies should therefore take into account novel biomarkers, such as microRNAs (miRNAs), high-mobility group box 1 (HMGB1) protein, and proteomic-based assays [26, 27]. MiRNAs are gaining in interest as potential noninvasive serum biomarkers in several cancers, including MM. For example, miR-126 correlated well with SMRP concentrations in MM patients and the combination was proposed as an early detection biomarker [28]. Proinflammatory cytokine HMGB1 is increased in serum of MM patients, since it drives and sustains MM development and it was proposed as an early detection biomarker and a possible drug target in MM [29, 30]. Proteomic-based assays simultaneously measuring several proteins could also help to identify novel biomarkers in MM as studies have shown such proteomic signatures could be used in surveillance and diagnosis of MM [31, 32]. Our future perspective would therefore be to investigate if addition of miRNAs or other protein biomarkers to MMP9 plasma levels and *MMP9* rs17576 A>G polymorphism may improve prognostic value of MMP9 in MM.

## 5. Conclusions

In conclusion, we have shown that MMP9 concentration correlates with ECOG performance status and that *MMP9* rs17576 influences MMP9 serum concentration in MM patients. However, we believe that MMP9 could have a higher significance as a composite biomarker with other novel biomarkers that are investigated in MM.

## Figures and Tables

**Table 1:** Patients' characteristics (*N* = 110) and their association with serum MMP9 before treatment.

Characteristic		*N* (%)	MMP9 before treatment (ng/ml) Median (25%-75%)	*P*
Gender	Male	89 (80.9)	707.8 (502.3-1224.7)	0.651^a^
	Female	21 (19.1)	683.5 (404.1-1231.8)	

Age	Median (25%-75%) (years)	66 (59.8-72.3)	Spearman's rho = −0.036	0.410^b^

Stage	I	12 (10.9)	595.8 (502.6-1412.1)	0.982^a^
	II	22 (20.0)	729.0 (396.8-1216.0)	
	III	42 (38.2)	772.3 (452.6-1277.5)	
	IV	27 (24.5)	698.5 (548.9-1207.2)	
	Peritoneal	7 (6.4)	757.4 (632.5-1511.6)	

Histological type	Epithelioid	81 (73.6)	757.4 (497.6-1213.2)	0.830^a^
	Biphasic	15 (13.6)	683.5 (393.3-1408.9)	
	Sarcomatoid	11 (810.0)	641.3 (483.4-1178.1)	
	Not characterized	3 (2.7)	705.7 (698.5-705.7)	

ECOG performance status	0	13 (11.8)	466.7 (383.2-842.5)	**0.010** ^a^
	1	56 (50.9)	682.0 (431.5-1088.4)	
	2	41 (37.3)	1026.5 (579.9-1443.0)	

CRP	Median (25%-75%) (mg/l)	17.5 (6.3-54.3) [6]	Spearman's rho = 0.067	0.498^b^

LDH	Median (25%-75%) (*μ*kat/l)	2.6 (2.2-2.9) [6]	Spearman's rho = −0.066	0.505^b^

Asbestos exposure	Not exposed	24 (21.8)	644.1 (502.6-1238.1)	0.625^a^
	Exposed	86 (78.2)	749.1 (497.1-1216.0)	

Smoking	Nonsmokers	61 (56.0) [1]	707.8 (406.6-1293.5)	0.732^a^
	Smokers	48 (44.0)	687.7 (558.0-1199.9)	

Pain	No	50 (45.9) [1]	641.3 (497.1-1243.9)	0.349^a^
	Yes	59 (54.1)	800.2 (546.1-1242.3)	

Weight loss	No	32 (29.6) [2]	868.9 (567.9-1175.4)	0.699^a^
	Yes	76 (70.4)	682.0 (485.9-1357.7)	

Numbers in denote missing data. ^a^Calculated using the Mann-Whitney or Kruskal-Wallis test. ^b^Calculated using Spearman's rho. CRP: C-reactive protein; ECOG: Eastern Cooperative Oncology Group; LDH: lactate dehydrogenase.

**Table 2:** Association of MMP9 serum levels with treatment response.

Serum MMP9	CR+PR Median (25-75%) (ng/ml)	SD+PD Median (25-75%) (ng/ml)	OR (95% CI)	*P*	OR (95% CI) adj	*P* adj
Before treatment	757.4 (546.1-1175.5)	683.5 (483.4-1242.3)	1.014 (0.938-1.095)	0.728	1.032 (0.951-1.118)	0.452
After treatment	449.3 (226.4-763.3)	440.5 (269.2-640.3)	1.036 (0.925-1.160)	0.542	1.092 (0.983-1.236)	0.164
Change after treatment	-299.4 (-603.1 to -8.5)	-286.3 (-648.5 to -74.2)	1.012 (0.925-1.107)	0.797	1.020 (0.925-1.124)	0.696

Odds ratios (ORs) calculated for an increase of MMP9 levels for 100 ng/ml. Adj: adjusted for C-reactive protein; CR: complete response; PR: partial response; SD: stable disease; PD: progressive disease.

**Table 3:** Association of *MMP9* SNPs with serum MMP9 levels before treatment.

Genotype	*N* (%)	Serum MMP9 (ng/ml) Median (27%-75%)	*P*
rs17576 c.836A>G, p.Gln279Arg			**0.041** ^a^
AA	45 (41.7) [2]	669.8 (429.4-973.2)	Reference
AG	55 (50.9)	698.5 (530.2-1244.5)	0.139
GG	8 (7.4)	1313.6 (689.4-1591.6)	**0.013**
AG+GG	63 (58.3)	869.6 (546.1-1420.2)	0.053
rs2250889 c.1721C>G, p.Arg574Pro			
CC	99 (91.7) [2]	753.8 (530.2-1219.1)	Reference
CG	9 (8.3)	503.0 (430.0-1084.7)	0.232
rs17577 c.2003G>A, p.Arg668Gln			0.146^a^
GG	76 (69.7) [1]	661.8 (435.9-1130.8)	Reference
GA	30 (27.5)	1035.5 (538.6-1411.7)	0.187
AA	2 (2.8)	1207.2 (820.4-1207.2)	0.120
GA+AA	32 (30.3)	1053.5 (545.1-1414.5)	0.100
rs20544 c.∗3C>T			0.222^a^
CC	18 (16.7) [2]	981.0 (558.0-1441.2)	Reference
CT	54 (50.0)	721.5 (493.9-1225.5)	0.275
TT	36 (33.3)	687.7 (425.2-975.3)	0.093
CT+TT	90 (83.3)	702.1 (486.8-1176.1)	0.148

^a^Calculated using the Kruskal-Wallis test comparing all three genotypes. Numbers in denote missing genotype data.

**Table 4:** Association of serum MMP9 with time to progression (TTP) and overall survival (OS).

Serum MMP9	TTP				OS			
	HR (95% CI)	*P*	HR (95% CI) adj^a^	*P* adj^a^	HR (95% CI)	*P*	HR (95% CI) adj^b^	*P* adj^b^
Before treatment (ng/ml)	1.020 (0.981-1.061)^c^	0.309	1.020 (0.981-1.061)^c^	0.464	1.033 (0.985-1.084)^c^	0.180	1.027 (0.977-1.080)^c^	0.293
After chemotherapy (ng/ml)	1.025 (0.965-1.089)^c^	0.420	1.011 (0.948-1.077)^c^	0.748	1.027 (0.951-1.109)^c^	0.494	1.007 (0.929-1.092)^c^	0.868
Change after chemotherapy (ng/ml)	0.985 (0.942-1.029)^c^	0.488	0.981 (0.936-1.028)^c^	0.417	0.973 (0.923-1.025)^c^	0.300	0.966 (0.916-1.020)^c^	0.209
Change after chemotherapy—increase vs. decrease	0.689 (0.393-1.207)	0.193	0.681 (0.384-1.208)	0.189	0. 256 (0.306-1.276)	0.197	0.586 (0.286-1.198)	0.143
At disease progression (ng/ml)					0.997 (0.929-1.069)^c^	0.929	0.975 (0.900-1.056)^c^	0.532

^a^Adjusted for C-reactive protein and histological type. ^b^Adjusted for C-reactive protein. ^c^Hazard ratios (HRs) calculated for increase of MMP9 levels for 100 ng/ml.

## Data Availability

The data used to support the findings of this study are included within the article and supplementary information file.

## Supplementary Materials

Supplementary Materials Supplementary Table 1: association of MMP9 haplotypes with serum MMP9 levels before treatment.
