# Supplementary material for: Evaluation of Matrix Metalloproteinase 9 Serum Concentration as a Biomarker in Malignant Mesothelioma
Source: Dis Markers. 2019 May 2;2019:1242964. doi: 10.1155/2019/1242964 (PMC6525906; doi:10.1155/2019/1242964)
Supplement: Supplementary Materials — Supplementary Table 1: association of MMP9 haplotypes with serum MMP9 levels before treatment. [file 1242964.f1.pdf]

Supplementary table 1: Association of *MMP9* haplotypes with serum MMP9 levels before treatment.

**Haplotype**	**Estimated frequency**	**Estimated mean MMP9 (ng/ml) (95% CI)**	**Difference compared to reference haplotype (95% CI)**	**P**
ACGT	0.59	382.8 (312.6-453.1)	Reference	
GCGC	0.19	527.0 (386.5-667.5)	144.2 (-29.7-318.0)	0.104
GCAC	0.14	593.2 (388.9-797.6)	210.4 (-17.8-438.6)	0.071
ACGC	0.04	451.8 (-71.2-974.8)	69.0 (-459.4-597.3)	0.798
AGAC	0.03	358.1 (-689.5-1405.6)	-24.8 (-1071.3-1021.7)	0.963
AGGC	0.02	27.6 (-4450.2-4505.5)	-355.2 (-4835.7-4125.3)	0.877

The SNPs are ordered from the 5′- to 3′-end as follows: rs17576, rs2250889, rs17577, and rs20544
